# Phosphorylated AKT and MAPK expression in primary tumours and in corresponding metastases and clinical outcome in colorectal cancer patients receiving irinotecan-cetuximab

**DOI:** 10.1186/1479-5876-10-71

**Published:** 2012-04-10

**Authors:** Mario Scartozzi, Riccardo Giampieri, Elena Maccaroni, Alessandra Mandolesi, Simona Biagetti, Simona Alfonsi, Lucio Giustini, Cristian Loretelli, Luca Faloppi, Alessandro Bittoni, Maristella Bianconi, Michela Del Prete, Italo Bearzi, Stefano Cascinu

**Affiliations:** 1 Clinica di Oncologia Medica, AO Ospedali Riuniti-Università Politecnica delle Marche, Marche Via Conca, Ancona, 60020, Italy; 2 Scuola di Specializzazione in Oncologia, Università Politecnica delle Marche, Ancona, Italy; 3 Anatomia Patologica, Università Politecnica delle Marche, Ancona, Italy; 4 Oncologia Medica, Ospedale di Fermo, Fermo, Italy

**Keywords:** Phosphorylated AKT, Phosphorylated MAPK, Liver metastases, Cetuximab, Colorectal cancer, K-RAS

## Abstract

**Background:**

Clinical observations suggested that a non negligible proportion of patients, ranging from 40% to 70%, does not seem to benefit from the use of anti-EGFR targeted antibodies even in the absence of a mutation of the K- RAS gene. The EGFR pathway activation via the Ras-Raf-MAP-kinase and the protein-serine/threonine kinase AKT could determine resistance to anti-EGFR treatment.

**Methods:**

We tested the interaction between phosphorylated AKT and MAPK expression in colorectal tumours and corresponding metastases and global outcome in K-RAS wild type patients receiving irinotecan-cetuximab.

**Results:**

Seventy-two patients with histologically proven metastatic colorectal cancer, treated with Irinotecan and Cetuximab based chemotherapy, were eligible for our analysis.

In metastases pAKT correlated with RR (9% vs. 58%, p = 0.004), PFS (2.3 months vs.9.2 months p < 0.0001) and OS (6.1 months vs.26.7 months p < 0.0001) and pMAPK correlated with RR (10% vs., 47%, p = 0.002), PFS (2.3 months vs.8.6 months p < 0.0001) and OS (7.8 months vs.26 months p = 0.0004). At multivariate analysis pAKT and pMAPK in metastases were able to independently predict PFS. pAKT in metastases independently correlated with RR as well

**Discussion:**

pAKT and pMAPK expression in metastases may modulate the activity of EGFR-targeted antibodies. We could speculate that in patients with pAKT and pMAPK metastases expression targeting these factors may be crucial.

## Background

Monoclonal antibodies against the ligand-binding site of the epidermal growth factor receptor (EGFR) have been shown to improve global outcome of metastatic colorectal cancer patients [1-5]. The introduction of K-RAS mutational status for patients selection in this setting appeared to possess the necessary potential for a full translation into clinical practice of the concept of targeted therapy [5-7].

In fact although we are now able to exclude from anti-EGFR treatment patients with putative refractory colorectal tumours (i.e. those harboring a K-RAS mutant status), we are still unable to select responding patients among those without K-RAS mutations. Clinical observations suggested that a non negligible proportion of patients, ranging from 40% to 70%, does not seem to benefit from the use of anti-EGFR targeted antibodies even in the absence of a mutation of the K- RAS gene (i.e. K-RAS wild-type) [2,4-7].

Preclinical observations demonstrated that binding of specific ligands, such as the epidermal growth factor (EGF) and transforming growth factor α (TGF-α) to the EGFR, results in the dimerisation of the receptor with the subsequent initiation of the intracellular signalling pathways cascade. A major downstream signalling route is via the Ras-Raf-MAP-kinase. Activation of Ras initiates a multistep phosphorylation cascade that leads to the activation of MAPKs, ERK1, and ERK2, which ultimately regulate transcription of molecules involved in cell proliferation [8,9]. Another important target in EGFR signalling is phosphatidylinositol 3-kinase (P13K) and the downstream protein-serine/threonine kinase AKT. This latter protein kinase transduces molecular signals triggering crucial steps for cell growth and survival [8-10].

It has been also hypothesized that the activation of the downstream signalling pathway (AKT and MAPK) could be responsible for EGFR aberrant activity even in the absence of a detectable EGFR expression [11-14]. In this case targeting the receptor via monoclonal antibodies would probably be clinically irrelevant. In fact in non small cell lung cancer patients it has been suggested that TKIs responsiveness might be predicted by EGFR downstream proteins such as activated (phosphorylated) AKT [15,16]. However, data regarding the *in vivo* EGFR-driven molecular profile in colorectal cancer are conflicting and consequently at the present no speculations are possible about its role in determining resistance and/or sensitivity to EGFR-targeted drugs.

In a series of 28 metastatic colorectal patients treated with gefitinib monotherapy, biologic evaluation of total and activated EGR, activated AKT, MAP-kinase and Ki 67 on paired pre- and 1 week post- treatment tumour samples could not confirm a gefitinib-induced decreased expression of these molecular markers [17].

Moreover no significant correlation has been found between pAKT expression and clinical outcome in metastatic colorectal cancer patients treated with cetuximab [18]. However patients were not stratified for K-RAS status and therefore firm conclusions were not possible.

A further potential confounding factor in this setting is the evidence that AKT and MAPK expression in primary colorectal tumours may not correlate with the expression in corresponding metastases and therefore AKT and MAPK [19]. 

We tested the interaction between phosphorylated AKT and MAPK in primary colorectal tumours and corresponding metastases and clinical outcome in terms of response rate (RR), progression free survival (PFS) and overall survival (OS) in order to identify a group of K-RAS wild type patients more likely to benefit from EGFR-targeted treatment.

## Methods

### Patients selection

Patients with histologically proven metastatic colorectal cancer, treated with Irinotecan and Cetuximab based chemotherapy at three different Italian institutions (Ancona, Fermo, Fabriano) between January 2007 and January 2011 were eligible for our analysis. Tumour response was evaluated every 8 weeks by clinicians’ assessment and according to the Response Evaluation Criteria in Solid Tumours (RECIST).

This study was approved by Ethical committee AOU Ospedali Riuniti – Umberto I of our institution. All patients provided informed written consent.

Analysis on the corresponding metastatic site was performed only in case tumour tissue from surgical resection of metastases was available.

### K-RAS mutational analysis

Formalin-fixed and paraffin-included tumour samples were analyzed for KRAS exon 2 mutations, located within the codon 12 and 13. After the purification using QIAquick® PCR Purification kit, the PCR products were direct sequenced with Big Dye V1.1 Terminator Kit (Applied Biosystems, Foster City, CA, USA) and an ABI Prism 3100 DNA sequencer (Applied Biosystems).

### Immunohistochemical analysis

The expression of phospho-AKT (Ser437) and p44/42 MAP kinase, (*)* was evaluated with an immunohistochemistry technique on 5-μm-thick tissue section obtained from paraffin-embedded specimens fixed in 10% (v/v) neutral buffered formalin.

The sections were deparaffinised and hydrated by passing through xylene and a graded series of ethanol, followed by washing in distilled water.

The antigens were unmasked for phospho-AKT (Ser437) by heat treatment at 98°C 10 min, in EDTA buffer and for p44/42 MAP kinase by microwave treatment at 98°C 10 minutes, in a 10 mM citrate buffer, pH 6.0. After antigens retrieval tissues were blocked with 5% normal goat serum for 60 min.

Subsequently the sections were incubated either with Phospho-AKT (Ser437) antibody (1:50 dilution) or MAP kinase antibody (1:100 dilution) overnight at 4°C.

Consecutively immunostaining was performed by the avidin-biotin peroxidase complex technique () for 30 min. according to the manufacturer’s instructions and using 3′, 3′ diaminobenzidine (DAB, ) as a chromogen.

Subsequently, the slides were counterstained with Meyer’s haematoxylin for 1 min., dehydrated in a graded series of alcohol, treated with xylene and cover slipped.

Positive control of Phospho-AKT (Ser437) and p44/42 MAP kinase staining consisted was performed on paraffin-embedded human breast cancer in all runs. *(Data Sheet of Phospho-AKT (Ser437) and p44/42 MAP kinase antibodies)*.

Negative control for the validation of the Phospho-AKT (Ser437), p44/42 MAP kinase assay consisted on sections incubated with secondary alone without primary antibody in all runs. *(Data Sheet of Phospho-AKT (Ser437) and p44/42 MAP kinase antibodies).*

All slides were evaluated independently by two pathologists (I.B. and A.M.).

Phospho-AKT expression was detected as cytoplasmic and nuclear staining of neoplastic cells with various intensity. The intensity of Phospho-AKT (Ser473) reactivity was scored using a four-tier system: 0, no staining, 1 weak, 2 moderate 3 strong.

Positivity for expression Phospho-AKT (Ser473) was defined as citoplasmatic staining with score 2 and/or 3, negativity with score 0 and/or 1. Both the primary and metastatic neoplasm were considered positive when more than 1% of the tumour cells had score 2 and/or 3 [20].

p44/42 MAP kinase expression was detected as cytoplasmic and nuclear brown staining of neoplastic cells. The intensity of p44/42 MAP kinase reactivity was scored using a four-tier system as follows: 0: no staining, 1:weak, 2: moderate, 3:strong signal intensity The proportion of neoplastic cells showing a positive signal was scored by assessing on a scale of 0 to 1: 0 none; 0,1 less than one tenth; 0,5 less than one half, and 1,0 greater than one half. The intensity and proportion scores were then multiplied to give an H-score; tumours with a score equal to or higher than 1,0 were deemed positive [21,22].

### Statistical analysis

Statistical analysis was performed with the MedCalc package (MedCalc® v9.4.2.0).

The association between categorical variables, such as phosphorylated AKT and phosphorylated MAPK and clinical outcome parameters was estimated by chi-square test.

Survival distribution was estimated by the Kaplan-Meier method. Significant differences in probability of relapsing between the strata were evaluated by log-rank test. A significant level of 0.05 was chosen to assess the statistical significance.

For statistical analysis overall survival and progression-free survival were defined respectively as the interval between the start of cetuximab and irinotecan therapy to death or last follow-up visit and as the interval between the start of cetuximab and irinotecan therapy to clinical progression or death or last follow up visit if not progressed.

Cox multiple regression analysis was used to assess the role of clinical prognostic factors adjusted for those variables resulted significant at univariate analysis. Odds ratios for response were evaluated from multivariate logistic regression for those variables resulted significant at univariate analysis.

Tested variables included: age (<65 vs. ≥ 65 years), gender (female versus male), performance status (Eastern Cooperative Oncology Group performance score, 0–1 vs. ≥ 2) and number of metastatic sites (1 vs. ≥ 2), phosphorylated AKT and MAPK status in primary tumours and metastases.

## Results

Seventy-two patients were included in our study, 51 males (70%) and 21 females (30%). Median age at diagnosis was 65 years (range 36–80). Most of the patients (61%) were treated with the irinotecan-cetuximab combination and had more than 1 metastatic sites. Main clinical characteristics are summarised in Table 1. In 37 cases the corresponding metastatic sites (all liver metastases) were available for AKT and MAPK analysis (Table 2).

**Table 1:** Patients characteristics and main study results for AKT and MAPK immunohistochemistry in primary colorectal tumours

	**Whole Group (n = 72)**	**AKT positive (n = 31)**	**AKT negative (n = 41)**	***p*value**	**MAPK positive (n = 32)**	**MAPK Negative (n = 40)**	***p*value**
**Age (range)**	65 (36–80)	67 (41–80)	65 (36–78)	ns	65 (38–80)	66 (40–79)	ns
**Sex**							
Males	51 (70%)	20 (65%)	31 (65%)	ns	23 (72%)	28 (70)	ns
Females	21 (30%)	11 (35%)	10 (35%)	ns	9 (28%)	12 (30%)	ns
**ECOG PS**							
0–1	58 (81%)	27 (87%)	31 (76%)	ns	29 (90%)	29 (72%)	ns
2–3	14 (19%)	4 (13%)	10 (23%)	ns	3 (10%)	11 (28%)	ns
**Metastatic sites**							
1	21 (30%)	8 (26%)	13 (32%)	ns	10 (32%)	11 (27%)	ns
≥ 2	51 (70%)	23 (74%)	28 (68%)	ns	22 (68%)	29 (73%)	ns
**Previous lines of treatment**							
1	10 (14%)	4 (13%)	6 (15%)	ns	3 (9%)	7 (17%)	ns
≥ 2	62 (86%)	27 (87%)	35 (85%)	ns	29 (91%)	33 (83%)	ns
**Treatment**							
mFOLFIRI + Cetuximab	28 (39%)	10 (32%)	18 (44%)	ns	12 (37%)	16 (40%)	ns
Irinotecan + Cetuximab	44 (61%)	21 (68%)	23 (56%)	ns	20 (63%)	24 (60%)	ns
**Response Rate**							
PR	21 (29%)	5 (16%)	16 (39%)	ns	9 (28%)	12 (30%)	ns
SD	19 (26%)	8 (26%)	11 (27%)	ns	7 (22%)	9 (22%)	ns
PD	32 (45%)	18 (58%)	14 (34%)	ns	16 (50%)	19 (48%)	ns
**Survival**							
m PFS (months)	3.2	2.4	6.5	0.0006	3	6	ns
m OS (months)	17.7	7.8	26.7	<0.0001	11.2	26.2	ns

*ns* = not significant

*ECOG PS* = Eastern cooperative oncology croup performance score

*mFOLFIRI* = modified FOLFIRI (irinotecan 180 mg/sqm d1, 5FU bolus 400 mg/sqm d1, 5FU 2400 mg/sqm continuous infusion for 46 hrs;

*PR* = partial remission;

*SD* = stable disease;

*PD* = progressive disease;

*m PFS* = median progression-free survival;

*m OS* = media overall survival.

**Table 2:** Patients characteristics and main study results for AKT and MAPK immunohistochemistry in metastases

	**Mets Group (n = 37)**	**AKT positive (n = 23)**	**AKT negative (n = 14)**	***p*value**	**MAPK positive (n = 20)**	**MAPK Negative (n = 17)**	***p*value**
**Age (range)**	66 (36–80)	67 (36–79)	65 (38–80)	ns	65 (40–80)	66 (40–79)	ns
**Sex**							
Males	27 (73%)	15 (65%)	12 (85%)	ns	13 (60%)	14 (82)	ns
Females	10 (27%)	8 (35%)	2 (15%)	ns	7 (40%)	3 (18%)	ns
**ECOG PS**							
0–1	28 (76%)	17 (74%)	11 (78%)	ns	16 (80%)	12 (70%)	ns
2–3	9 (24%)	6 (26%)	3 (22%)	ns	4 (20%)	5 (30%)	ns
**Metastatic sites**							
1	10 (27%)	6 (26%)	4 (28%)	ns	5 (25%)	5 (29%)	ns
≥ 2	27 (73%)	17 (74%)	10 (72%)	ns	15 (75%)	12 (71%)	ns
**Previous lines** **of treatment**							
1	9 (24%)	5 (22%)	4 (28%)	ns	5 (25%)	4 (23%)	ns
≥ 2	28 (76%)	18 (78%)	10 (72%)	ns	15 (75%)	13 (77%)	ns
**Treatment**							
mFOLFIRI + Cetuximab	12 (32%)	6 (26%)	6 (43%)	ns	7 (35%)	5 (30%)	ns
Irinotecan + Cetuximab	25 (68%)	17 (74%)	8 (57%)	ns	13 (65%)	12 (70%)	ns
**Response Rate**							
PR	10 (27%)	2 (9%)	8 (58%)	0.004	2 (10%)	8 (47%)	0.002
SD	9 (25%)	6 (26%)	3 (21%)	ns	5 (25%)	4 (23%)	ns
PD	18 (48%)	15 (65%)	3 (21%)	0.001	13 (65%)	5 (30%)	0.006
**Survival**							
m PFS (months)	3.2	2.3	9.2	<0.0001	2.3	8.6	<0.0001
m OS (months)	12.6	6.1	26.7	<0.0001	7.8	26	0.0004

*ns* = not significant

*ECOG PS* = Eastern cooperative oncology croup performance score

*mFOLFIRI* = modified FOLFIRI (irinotecan 180 mg/sqm d1, 5FU bolus 400 mg/sqm d1, 5FU 2400 mg/sqm continuous infusion for 46 hrs;

*PR* = partial remission;

*SD* = stable disease;

*PD* = progressive disease;

*m PFS* = median progression-free survival;

*m OS* = median overall survival.

### AKT and MAPK results in primary colorectal tumours

AKT was positive in 31 primary colorectal tumours (43%) and negative in the remaining 41 patients (57%). MAPK was positive in 32 primary colorectal tumours (44%) and negative in 40 cases (56%). Clinical characteristics resulted well balanced across groups (Table 1).

**Figure 1:**
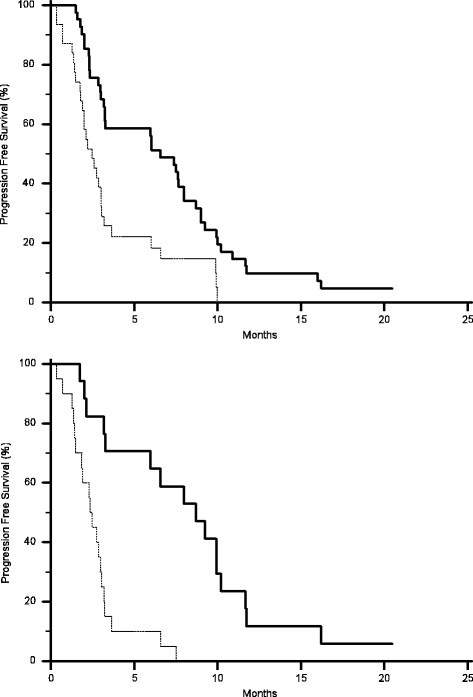
Kaplan-Meier curves for median progression free survival (PFS) of colorectal cancer patients treated with irinotecan and cetuximab showing phosphorylated AKT expression (-------) and without phosphorylated AKT expression (———) in either primary tumours (A, 2.4 months vs. 6.5 months, p = 0.0006) or metastases (B, 2.3 months vs. 9.2 months, p = < 0.0001).

**Figure 2:**
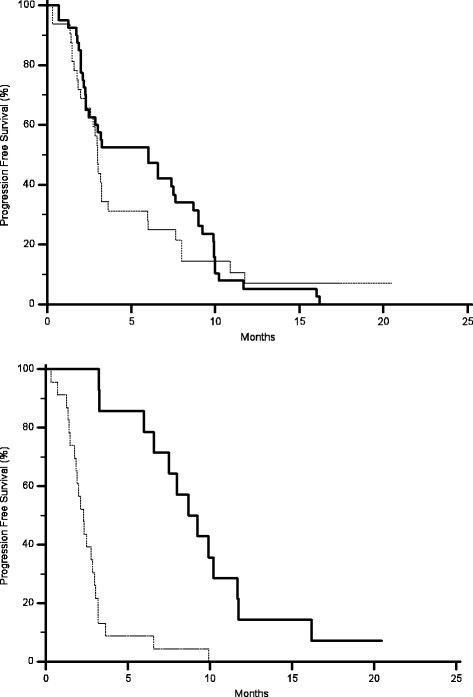
Kaplan-Meier curves for median overall survival (OS) of colorectal cancer patients treated with irinotecan and cetuximab showing phosphorylated AKT expression (-------) and without phosphorylated AKT expression (———) in either primary tumours (A, 7.8 months vs. 26.7 months, p < 0.0001) or metastases (B, 6.1 months vs. 26.7 months, p < 0.0001).

**Figure 3:**
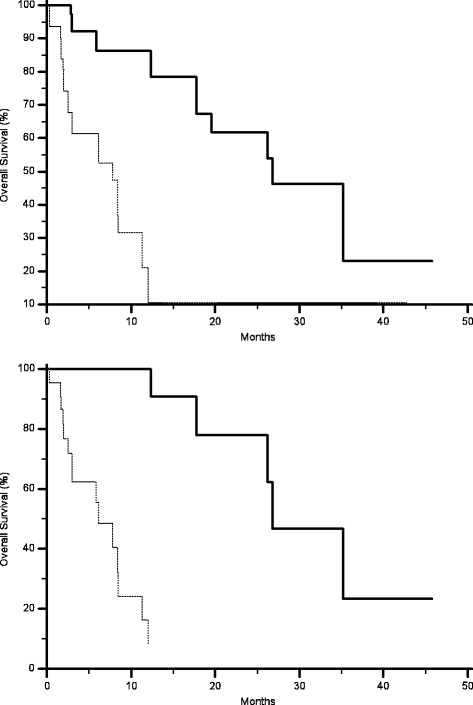
Kaplan-Meier curves for median progression free survival (PFS) of colorectal cancer patients treated with irinotecan and cetuximab showing phosphorylated MAPK expression (-------) and without phosphorylated MAPK expression (——) in either primary tumours (A, 3 months vs. 6 months, p = 0.6) or metastases (B, 2.3 months vs. 8.6 months, p = < 0.0001).

**Figure 4:**
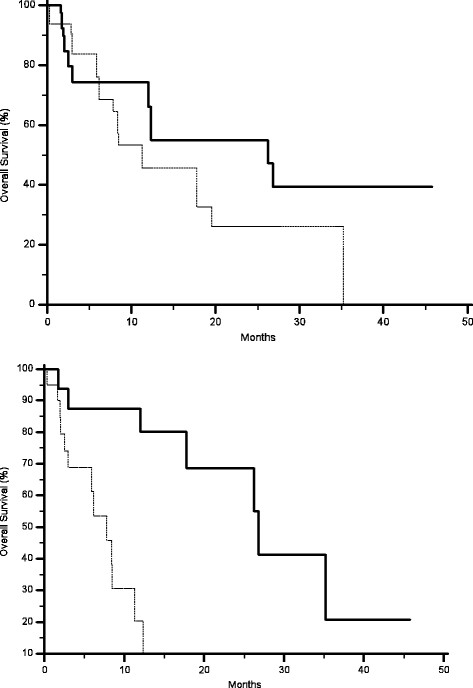
Kaplan-Meier curves for median overall survival (OS) of colorectal cancer patients treated with irinotecan and cetuximab showing phosphorylated MAPK expression (-------) and without phosphorylated MAPK expression (———) in either primary tumours (A, 11.2 months vs. 26.2 months, p = 0.1) or metastases (B, 7.8 months vs. 26 months, p = 0.0004).

AKT expression in primary tumours correlated with a statistically significant worse median PFS (2.4 months vs. 6.5 months, p = 0.0006) (Figure 1) and OS (7.8 months vs. 26.7 months, p < 0.0001) (Figure 2), without any significant correlation with RR. No significant correlation could be found between MAPK expression in primary tumours and either RR, median PFS or OS (Table 1) (Figures 3 and 4).

### AKT and MAPK results in corresponding metastases

Analysis on the corresponding metastatic site was performed in tumour tissue from surgical resection of metastases. All resections were performed at the time of diagnosis.

AKT was positive in 23 out of the 37 metastases available for analysis (62%) and negative in the remaining 14 (38%) (Table 2). AKT status in primary colorectal tumours was not concordant with AKT expression in metastases in 10 cases (27%). MAPK was positive in 20 metastases (54%) and negative in 17 cases (46%). MAPK status in primary colorectal tumours was not concordant with MAPK expression in metastases in 11 cases (29%).

AKT expression correlated with RR (9% vs., 58%, p = 0.004), median PFS (2.3 months vs.9.2 months p < 0.0001) (Figure 1) and median OS (6.1 months vs. 26.7 months p < 0.0001) (Figure 2). Analogously MAPK expression in metastases correlated with RR (10% vs., 47%, p = 0.002), median PFS (2.3 months vs.8.6 months p < 0.0001) (Figure 3) and median OS (7.8 months vs.26 months p = 0.0004) (Table 2) (Figure 4).

### Multivariate analysis

Tested variables were: AKT expression in primary tumours, AKT expression in metastases and MAPK expression in metastases.

Multivariate logistic regression analysis for response showed that AKT expression in metastases independently correlated with response rate (OR = 0.13, 95%CI 0.018–0.9, p = 0.04), whereas neither AKT in primary tumours nor MAPK in metastases were independently associated with response.

Multivariate Cox regression analysis indicated that AKT expression in metastases and MAPK expression in metastases both correlated with median progression free survival (HR = 0.63, 95%CI 0.42–0.86, p = 0.0007 and HR = 0.49, 95%CI 0.28–0.95, p = 0.002 respectively). AKT expression in primary tumours did not independently correlate with PFS. None of the tested variables independently correlated with median overall survival (Table 3).

**Table 3:** Multivariate analysis results for response rate (RR) and progression-free survival (PFS)

	**Metastases**			
	**AKT Positive**	**AKT negative**	**MAPK Positive**	**MAPK Negative**
**Response Rate (%)**	9%	58%	10%	47%
**Multivariate OR (95%CI)**	0.13 (0.018–0.9)		0.26 (0.03–1.95)	
**Logistic regression*p*value**	0.04		0.9	
**Median PFS (months)**	2.3	9.2	2.3	8.6
**Multivariate HR (95%CI)**	0.63 (0.42–0.86)		0.49 (0.28–0.95)	
**Cox regression*p*value**	0.0007		0.002	

## Discussion

The introduction of K-RAS testing for colorectal cancer patients offered the long-awaited clinical opportunity to exclude resistant tumours from the treatment with anti-EGFR antibodies. However a not negligible proportion of colorectal tumours, ranging from 40 to 70% in different series, is still refractory to this therapeutic approach even in presence of a K-RAS wild type status [2-5]. Unfortunately to date it is not possible to further characterize, either biologically or clinically, this particular group of patients, who, in the end, receive an ineffective therapy at a cost of unwanted side-effects.

Many biological factors have been investigated with the aim to better identify responding patients in this setting, however initial observations were often conflicting and limited to a small proportion of patients thus precluding their effective use into the clinical practice [23-28]. The example of b-RAF is clearly emblematic. In fact preliminary data about the role of b-RAF mutational status seemed initially promising for a straightforward application in the clinical practice, but a large subsequent analysis from the CRYSTAL trial demonstrated that b-RAF although possessing a possible prognostic role had no predictive value [29,30].

In our analysis we suggested that activated AKT and MAPK expression in liver metastases could represent possible molecular determinants for the prediction of clinical outcome in K-RAS wild type colorectal cancer patients receiving irinotecan-cetuximab. These observations are in accordance with the biological assumption that the activation of the downstream signalling pathway (pAKT and pMAPK) could be responsible for EGFR aberrant activity independently from the receptor itself ultimately making treatment strategies targeting the EGFR ineffective. A prior study in colorectal cancer patients could not confirm any correlation between pAKT expression in primary tumours or metastases and either response or survival [18]. However in this analysis the role of phosphorylated AKT was assessed irrespectively of K-RAS status and this may represent a possible confounding factor for data interpretation. An analysis presented by Baba et al. showed that pAKT expression was correlated to good prognosis in colorectal cancer patients. In this latter study the analysis was conducted on primary tumours and no information was available about the treatment administered to the patients. Taken together these and our observations seem then to suggest that more than a prognostic factor pAKT is a predictive factor in presence of anti-EGFR inhibitors.

A further relevant finding from our analysis is that only AKT and MAPK status in metastases is relevant for the prediction of patients outcome. The variation (or conservation) of the primary colorectal tumours molecular profile in distant metastases is a current, but unresolved issue in molecular and clinical oncology. Published analyses in colorectal cancer patients indicated that the mutational status of some molecular determinants such as K-RAS and BRAF is almost completely unaltered from primary to corresponding metastases [31]. On the contrary other biological markers such as EGFR or PTEN, for example, demonstrated a significant variation [18,32].

A discordant pAKT and pMAPK expression in primary colorectal tumours and metastases has been previously suggested. A shift in pAKT and pMAPK expression from primary to metastases has been in fact reported in as much as 30% of all cases [18,19]. In the present study a non concordant status between primary tumours and metastases for AKT and MAPK was noticed respectively in 27% and 29% of examined cases thus confirming previous reports. More importantly our data demonstrated that this different expression pattern had a biological effect in modulating the activity of EGFR targeted antibodies, thus reinforcing the hypothesis that when treating metastatic disease with molecularly targeted agents attention should be paid to the biological markers expression profile in metastases.

## Conclusions

We could speculate that in colorectal tumours with pAKT and pMAPK expression a therapeutic strategy targeting these molecular factors may be more appropriate than anti-EGFR therapies. However these assumptions should be demonstrated before a possible application into the clinic.

We believe that our findings along with others already reported by other groups may help further composing the molecular mosaic of K-RAS wild type colorectal cancer patients especially in view of a prospective validation.
